# Phylogeny in Defining Model Plants for Lignocellulosic Ethanol Production: A Comparative Study of *Brachypodium distachyon*, Wheat, Maize, and *Miscanthus* x *giganteus* Leaf and Stem Biomass

**DOI:** 10.1371/journal.pone.0103580

**Published:** 2014-08-18

**Authors:** Till Meineke, Chithra Manisseri, Christian A. Voigt

**Affiliations:** Phytopathology & Biochemistry, Biocenter Klein Flottbek, University of Hamburg, Hamburg, Germany; University of Massachusetts Amherst, United States of America

## Abstract

The production of ethanol from pretreated plant biomass during fermentation is a strategy to mitigate climate change by substituting fossil fuels. However, biomass conversion is mainly limited by the recalcitrant nature of the plant cell wall. To overcome recalcitrance, the optimization of the plant cell wall for subsequent processing is a promising approach. Based on their phylogenetic proximity to existing and emerging energy crops, model plants have been proposed to study bioenergy-related cell wall biochemistry. One example is *Brachypodium distachyon*, which has been considered as a general model plant for cell wall analysis in grasses. To test whether relative phylogenetic proximity would be sufficient to qualify as a model plant not only for cell wall composition but also for the complete process leading to bioethanol production, we compared the processing of leaf and stem biomass from the C_3_ grasses *B. distachyon* and *Triticum aestivum* (wheat) with the C_4_ grasses *Zea mays* (maize) and *Miscanthus* x *giganteus*, a perennial energy crop. Lambda scanning with a confocal laser-scanning microscope allowed a rapid qualitative analysis of biomass saccharification. A maximum of 108–117 mg ethanol·g^−1^ dry biomass was yielded from thermo-chemically and enzymatically pretreated stem biomass of the tested plant species. Principal component analysis revealed that a relatively strong correlation between similarities in lignocellulosic ethanol production and phylogenetic relation was only given for stem and leaf biomass of the two tested C_4_ grasses. Our results suggest that suitability of *B. distachyon* as a model plant for biomass conversion of energy crops has to be specifically tested based on applied processing parameters and biomass tissue type.

## Introduction

The increasing global energy demand leads to an elevated consumption of fossil energy resources, which is not only associated with the observed climate change [1] but also a with a growing threat for sensitive ecosystems due to expanded explorations for fossil energy sources [2]–[4]. One strategy to face this challenge is the substitution of fossil by renewable energy sources. In the sector of transportation, liquid fossil fuels have a predominant role. Here, second generation biofuels from lignocellulosic feedstock have the potential for an extended substitution in the near future [5]. A main source of lignocellulosic feedstock could be stover and straw from field crops, which would mainly derive from *Zea mays* (maize) and *Triticum aestivum* (wheat) in temperate climates, where these two crops are predominantly cultivated in agriculture according to the 2012 FAO statistics of crop production [6]. However, to meet the expected demand on lignocellulosic feedstock and to improve a sustainable production of biomass, an extended cultivation of energy crops is a promising solution [7]. In terms of sustainability, perennial C_4_ grasses are preferred low-input energy crops because they can recycle their nutrients and store them in their rhizomes in the non-growing season, which results in a minimized fertilizer input. In addition, they have a reduced requirement for herbicide application because of fast growth, providing habitats for animals, and reducing soil erosion with their extended, permanent root systems [8]–[11]. Among the group of perennial C_4_ grasses, *Miscanthus* x *giganteus* not only meets the mentioned criteria for sustainable biomass production [12] but also proved to be the most productive energy crop under temperate climate conditions [13]–[15]. Hence, the calculated lignocellulosic ethanol yield per ha cropland for *M.* x *giganteus* was higher than in other designated energy crops including *Z. mays* and *Panicum virgatum* (switchgrass) [14].

However, the efficiency of lignocellulosic ethanol production is restricted by the recalcitrance of the cell wall, which results in a relatively low saccharification. Main factors that determine biomass saccharification are cellulose crystallinity [16]–[18] but also lignin and hemicellulose content [19], [20]. Apart from modifying and adapting different biomass pretreatment methods, the optimization of the cell wall for improved saccharification is a strategy to overcome biomass recalcitrance. Whereas different genetic tools and their application have already been established for major crops like *T. aestivum* and *Z.mays*, none of these tools are available for *M.* x *giganteus*. Therefore, the wild grass *Brachypodium distachyon* with its relatively small and sequenced diploid genome, efficient transformation, easy cultivation, and fast generation cycle has been proposed as a suitable model to test modified or engineered cell walls for altered saccharification. However, whereas genomic and cell wall composition studies already revealed the suitability of *B. distachyon* as a model for other grasses [21]–[25], comparative analyses of the complete process starting from biomass pretreatment via saccharification efficiency to fermentation leading to ethanol production have not been performed.

Our study aimed to test whether phylogenetic relation could be used as a marker for similarities in the processing of biomass for ethanol production with a special focus on *B. distachyon* and its qualities as a model plant for energy crops regarding ethanol production via fermentation. In case phylogenetic proximity would correlate with the process of biomass fermentation, we expected that results from *B. distachyon* would reveal highest similarity to *T. aestivum*, which are both C_3_ grasses, and not to the C_4_ grasses *Z. mays* and *M.* x *giganteus*. We distinguished between leaf and stem biomass because we identified a specific requirement to establish a model plant for stem biomass. In general, biomass from perennial grasses like *M.* x *giganteus* mainly derives from stems because it is typically harvested in early spring when most leaves have fallen off and the water content is low. We cultivated all plants and performed all experiments under the same conditions to ensure that observed differences or similarities would not be based on the experimental setup but on cell wall characteristics.

Our results revealed that phylogenetic relation cannot be regarded as a general marker for similarities in the processing of biomass for bioethanol production because other factors like biomass pretreatment as well as tissue type of the biomass seem to have an additional influence.

## Materials and Methods

### Plant material

*B. distachyon* (inbred line Bd21 [26]), *T. aestivum* (cultivar Nandu, Lochow-Petkus, Bergen-Wohlde, Germany), *M.* x *giganteus*, and *Z. mays* (inbred line A188 [27]) were all cultivated in two parts of soil (Einheitserdewerk Uetersen, Germany, ED 73+10% sand) and one part of sand under greenhouse conditions with an additional light supply to provide 16 h light if required. Plant material that reached its final developmental stage due to complete, natural senescence with subsequent drying for 2 weeks without irrigation was harvested from *B. distachyon* after 4 month, from *T. aestivum* and *Z. mays* after 6 month, and from *M.* x *giganteus* after 1 year. Plant material was manually harvested and separated into stem biomass, including all nodes, and leaf biomass, comprising blades and sheaths. Spikes and spikelets from *B. distachyon* and *T. aestivum* as well as cobs from *Z. mays* were not used in this study. After harvest, leaf and stem biomass was dried at 50°C for 2 days. Biomass was homogenized with a mill fitted with a 1.5 mm mesh screen for its use in pretreatment and fermentation experiments and with a 0.2 mm mesh screen for compositional analysis.

### Preparation of alcohol insoluble residue

Milled plant biomass was processed as described by Arora et al. [28] with the modifications as described by Ellinger et al. [29] to receive alcohol insoluble residue (AIR) and destarched AIR, which was used for lignin, cellulose, and hemicellulose quantification.

### Lignin quantification using acetyl bromide

The lignin content of destarched AIR was determined with a modified acetyl bromide method [28]. Lignin contents were determined with the absorbance at 280 nm and calculated with an extinction coefficient of 17.747 g^−1^·L·cm^−1^ for maize leaf and stem, 17.377 g^−1^·L·cm^−1^ for *B. distachyon* leaf and stem, 19.808 g^−1^·L·cm^−1^ for wheat leaf and 17.542 g^−1^·L·cm^−1^ for wheat stem [30]. For *M.* x *giganteus* leaf and stem, a specific extinction coefficient of 17.78 g^−1^·L·cm^−1^ was used for calculation [31].

### Cellulose and hemicellulose quantification using phenol/sulfuric acid

Destarched AIR biomass was used for quantification of cellulose and hemicellulose following a modified protocol of DuBois et al. [32]. For determination of the total cell wall sugar amount, 5 mg of AIR samples were mixed with 50 µL of 72% (w/w) sulfuric acid and incubated at 30°C for 1 h. After cooling down, these solutions were diluted with water to 4% (w/w) sulfuric acid and again incubated at 120°C for 1 h. Supernatants obtained after centrifugation at 13,000× *g* for 5 min were diluted 1∶400 with water. 0.5 mL of these dilutions were mixed with 0.3 mL 5% phenol and 1.8 mL sulfuric acid and incubated at room temperature for 20 min. Absorbance of these solutions were measured at 480 nm. For determination of the hemicellulose amount, 5 mg of AIR samples were treated with trifluoro acetic acid (TFA, 2 M) at 120°C for 1 h. This treatment hydrolyzes non-cellulosic polysaccharides but does not affect cellulose [33]. TFA hydrolyzates were completely dried using a speedvac concentrator (Savant, USA) at 32°C under vacuum. Monosaccharides liberated after TFA hydrolysis were redissolved in 1 mL of water. After centrifugation at 13,000× *g* for 5 min, 0.5 mL of 1∶200-diluted supernatants were mixed with 0.3 mL 5% phenol and 1.8 mL sulfuric acid and incubated at room temperature for 20 min. Absorbance of these solutions were measured at 480 nm. For the preparation of standards, 5–25 µg glucose and xylose in 0.5 mL total volume were mixed with 0.3 mL 5% phenol and 1.8 mL sulfuric acid and incubated at room temperature for 20 min. Amounts of hemicellulose were quantified with xylose as standard. Cellulose amounts were quantified by subtraction of the amount of hemicellulose from the calculated values from total cell wall sugar amount using the glucose standard.

### Monosaccharide composition by high-performance anion exchange chromatography with pulsed amperometric detection (HPAEC-PAD)

The non-cellulosic monosaccharide composition of leaf and stem cell walls before and after pretreatment and fermentation was analyzed by HPAEC-PAD on an ICS-5000 system (Dionex, USA) equipped with electrochemical detector and a CarboPac PA 20 column (3×150 mm, Dionex, USA), according to the description in Ellinger et al. [29].

### Pretreatment and fermentation of plant biomass

7.5 g milled leaf and stem biomass were mixed with 42.5 mL 1.75% (v/v) sulfuric acid and autoclaved for 1 h at 120°C in 300 mL fermentation reactors (DASGIP, Germany). 20 mL of 10× YP Broth (200 g peptone and 100 g yeast extract per liter) were added to the fermentation reactors and volume was adjusted to 200 mL with water. The 4× parallel fermenter (DASGIP) was programmed to pH 5.0 at 30°C with 600–900 rpm. Samples (2×2 mL) were taken when these values were reached. The fermentation broth was inoculated with 1 mL of an overnight grown *Saccharomyces cerevisiae* culture (strain MaV203, Life Technologies, Germany). Samples (2×2 mL) were taken every 24 h. In experiments with additional enzymatic pretreatment, the thermo-chemical pretreated biomass (200 mL as described above) was mixed with 1.875 mL of the enzyme mixture Accellerase 1500 (Genencor, Netherlands) according to the manufactures instructions. The fermentation broth was stirred for 24 h at 55°C. After samples were taken (2×2 mL), fermenters were inoculated with *S. cerevisiae* (as described above), which generally only ferments hexoses. The fermentation process was monitored and controlled with the DASGIP Control 4.0 software. Samples were centrifuged at 13,000 rpm for 5 min. Supernatants were filtered through 0.44 µm PTFE-filters. Glucose as well as ethanol concentrations were analyzed as described by Bonn and Bobleter [34] on an ICS-5000 system (Dionex) with a HPX 87H column (Bio-Rad, USA, mobile phase 0.005 M H_2_SO_4_, flow rate 0.6 mL·min^−1^, column temperature: 50°C, refractive index detector). Residues were used for lambda scanning in laser confocal fluorescence microscopy and for cell wall analysis HPAEC-PAD.

### Lambda scanning using confocal laser-scanning microscopy

Milled, untreated biomass, diluted acid pretreated biomass, diluted acid and enzyme pretreated biomass as well as fermented biomass after 96 h of fermentation (both pretreatments) were analyzed with a LSM 780 confocal laser-scanning microscope (Zeiss, Germany). For the rapid qualitative analysis of biomass saccharification, confocal lambda scanning was applied with a 405 nm diode laser and a 561 nm diode-pumped solid-state (DPSS) laser for excitation. Emission spectra were acquired with a meta-detector in the range of 411–691 nm [35]. Lambda scans were processed with integral functions of the ZEN 2010 (Zeiss) operating software. For each scan, three regions of the cell wall were manually selected and the relative intensities for each wavelength were measured.

### Phylogenetic analysis

DNA sequences from the second intergenic spacer (ITS2) of nuclear ribosomal DNA were aligned using the Clustal Omega [36] multiple sequence alignment tool provided by the EMBL European Bioinformatics Institute (http://www.ebi.ac.uk/tools/msa/clustalo/). Manual corrections of initial alignment followed guidelines of Kelchner [37]. PHYLIP interleaved alignment output format of the Clustal Omega online tool was used to generate a phylogenetic tree using the online phylogenetic tree drawing application Phylodendron (version 0.8 d; http://iubio.bio.indiana.edu/treeapp/). ITS2 sequence information previously used to generate phylogenetic trees was obtained from GenBank: *M*. x *giganteus* [AJ426562], *Z. mays* [AF019811], *Sorghum bicolor* (sorghum) [SBU04789], *Saccharum officinarum* (sugarcane) [AY116284], *T. aestivum* [FM998919], *Hordeum vulgare* (barley) [AF438194], *B. distachyon* [AF303399], *Oryza sativa* (rice) [JN402189], *Secale cereale* (rye) [AF303400], *Arabidopsis thaliana* [AJ232900], *Solanum lycopersicum* (tomato) [AJ300201], *Glycine max* (soybean) [AJ011337].

### Statistical analysis

Descriptive statistics including the mean and the standard error of the mean (SE) along Tukey range test for multiple comparison procedure in conjunction with an ANOVA were used to determine significant differences. *P*<0.05 was considered significant.

### Principal component analysis (PCA)

Separate two-factor PCA and associated PC loadings for factor 1 and 2 for leaf and stem biomass with thermo-chemical pretreatment and combined thermo-chemical and subsequent enzymatic pretreatment were calculated with the statistical data analysis software STATISTICA (version 9, Statsoft, Germany). In analogy to constant-sum normalization [38], PCA parameters of a specific plant species and biomass pretreatment were introduced as relative amounts (g·g^−1^ dry biomass) and set to the sum of 1 to achieve an internal normalization between the different datasets. PCA parameters used for calculation were relative amounts of lignin, cellulose, and hemicellulose, relative amounts of glucose deriving from the hemicellulose fraction, relative amounts of hydrolyzed glucose from biomass, and relative amounts of produced ethanol.

## Results and Discussion

### Biomass composition

**Figure 1:**
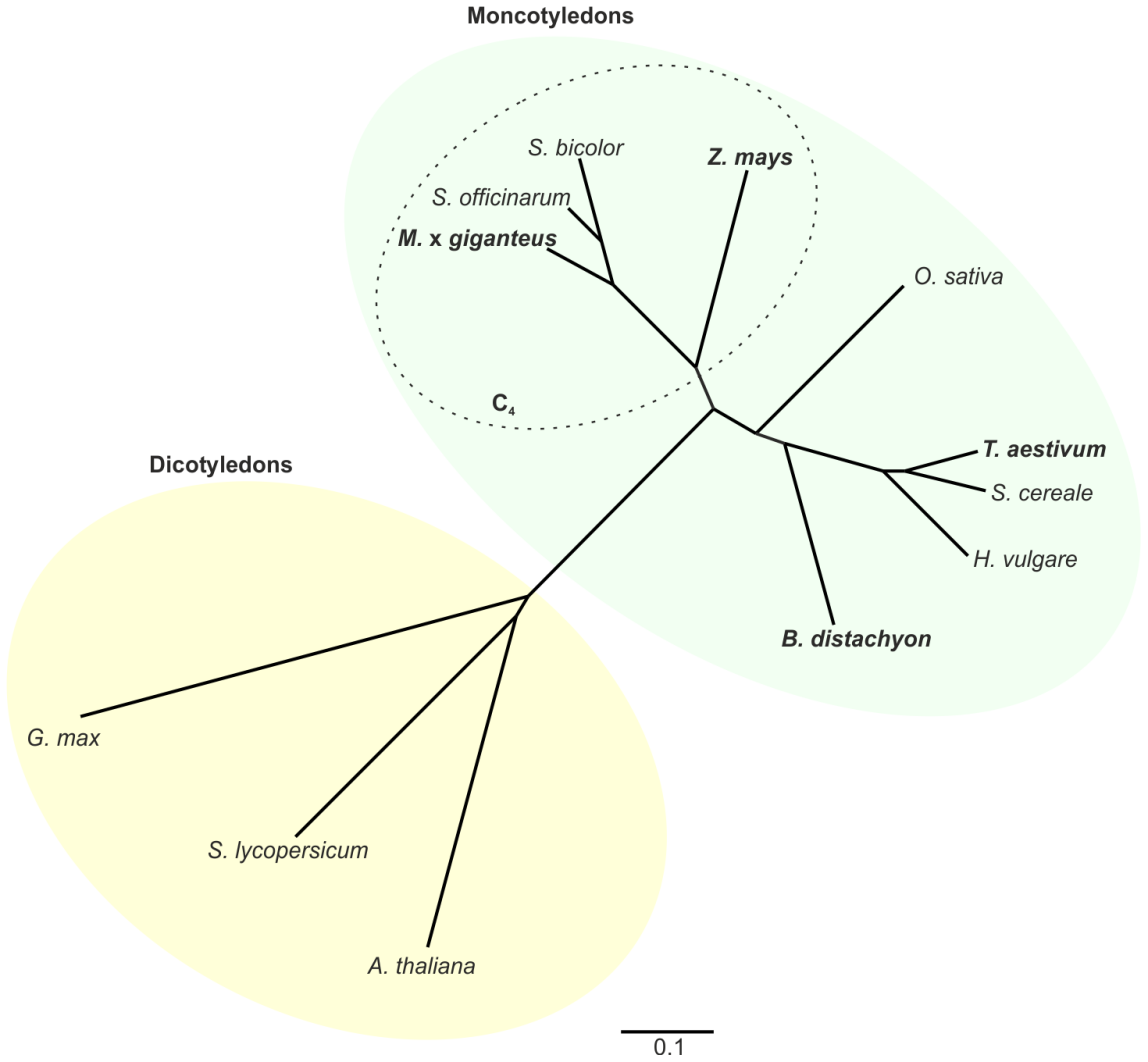
Phylogenetic relation of tested plant species. Phylogenetic tree based on the nucleotide alignment of the ITS2 region of ribosomal DNA of the C_4_ grasses *Z. mays* (maize), *S. officinarum* (sugarcane), *S. bicolor* (sorghum), and *M.* x *giganteus*, the C_3_ grasses *O. sativa* (rice), *T. aestivum* (wheat), *H. vulgare* (barley), *S. cereale* (rye), and *B. distachyon*, and the C_3_ dicots *G. max* (soybean), *S. lycopersicum* (tomato), and *A. thaliana*, which were used as an outgroup. Bold: plant species used in this study, dotted line: plant species with a C_4_ carbon fixation.

To test the application of *B. distachyon* as model plant for biomass conversion, we compared cell wall composition, saccharification, and lignocellulosic ethanol production of *B. distachyon* leaf and stem biomass with biomass of the crop *T. aestivum*, which is a C_3_ grass like *B. distachyon*, and the C_4_ grasses *Z. mays* and *M.* x *giganteus.* We generated a phylogenetic tree of the plant species used in this study and related crop species based on the alignment of the ITS2 nuclear ribosomal DNA spacer region (Fig. 1). As expected from previous phylogenetic analyses of the respective plant species, the C_4_ grasses *M*. x *giganteus*, *Z. mays*, *S. biocolor*, and *S. officinarum* formed a group that separated from the group of the C_3_ grasses *B. distachyon*, *T. aestivum*, *H. vulgare*, *S. cereale*, and *O. sativa*. Within these two groups, phylogenetic distances resembled those of previous studies where *M.* x *giganteus* revealed higher phylogenetic relation to *S. biocolor* and *S. officinarum* than to *Z. mays* [39], and *B. distachyon* a higher phylogenetic relation to the small grain cereals *T. aestivum*, *H. vulgare*, and *S. cereale* than to *O. sativa* [40]. The dicotyledonous model plant *A. thaliana* as well as the dicotyledonous crops *S. lycopersicum* and *G. max* outgrouped due to the relatively high phylogenetic distance to the monocotyledonous grasses [41].

**Figure 2:**
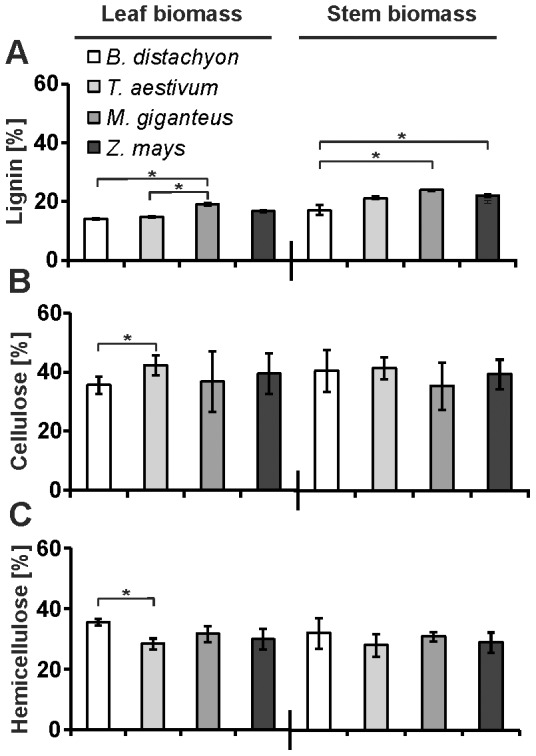
Cell wall composition of leaf and stem biomass. Milled leaf and stem samples from senesced, dry plants were used for the determination of the three major cell wall polymers (**A**) lignin, (**B**) cellulose, and (**C**) hemicelluloses. Values represent the relative amount of each polymer. *P<0.05 by Tukey's test. Error bars represent SE, and *n* = 3.

In our study, we started to determined the cellulose, hemicellulose, and lignin content of the harvested, naturally senesced biomass after an additional drying process (Fig. 2). In *Z. mays* stem biomass, we determined a relative cellulose content of 40%, which was only slightly higher than previously reported [42] whereas the hemicellulose content was slightly decreased, 29% compared to 33% [42]. In *M.* x *giganteus*, the cellulose content of stem biomass, which ranged from 42% to 45% in previous reports [31], [42], was reduced (36%) whereas we observed an increase in the hemicellulose content, 31% compared to the reported 27% [42] (Fig. 2B,C). The lignin content of 22% in *Z. mays* stem biomass (Fig. 2A) resembled the value that was previously reported (21%) [43]. In that study, the lignin content was also determine by the acetyl bromide method whereas amounts of lignin were reported to be lower in those studies where Klason lignin was determined [44], [45]. This contributes to the observation that values for the lignin content deriving from different methods are not directly comparable [30]. In addition, the lignin content in *Z. mays* stem biomass was strongly dependent on the tested inbred line and growing conditions [44], [45]. Whereas we used biomass from the *Z. mays* inbred line A188 grown under greenhouse conditions, other studies mainly refer to biomass from different, field grown *Z. maize* cultivars.

In *M.* x *giganteus* stem biomass, the lignin content was reduced compared to a previous report from field grown *M.* x *giganteus* (24% compared to 27% [31]) (Fig. 2A). Whether reduction of lignin content in *M*. x *giganteus* comparing greenhouse and field grown plants would indicate general differences in modifying cell wall composition in response to different growing conditions in this C_4_ grass, could be tested in comparative growth experiments. Regarding general cell wall composition of the analyzed C_3_ grasses, we observed a higher lignin content in *B. distachyon* stem biomass than previously reported, 17% compared to a reported content of 14% [43], but no differences in cellulose and hemicellulose content [43]. In *T. aestivum*, the cellulose content with 42% was in the range of 35% to 48% as previously reported [42], [46]. Also the hemicellulose content with 28% was in the range as indicated in previous studies (23% [42] to 28% [46]). The lignin content (21%) was also similar to previously reported amounts [30], [42] (Fig. 2).

Whereas it was possible to compare our data of the cell wall composition of stem biomass with existing data in literature for all tested plant species, comparable data of the cell wall composition were not available for leaf biomass. Therefore, our data from leaf biomass would represent a new reference for the cell wall composition of senesced, dry leaf biomass.

Comparing the content of the cell wall components cellulose and hemicellulose between the different plant species, we determined a significant difference in cellulose and hemicellulose content in leaf biomass only between the model grass *B. distachyon* and *T. aestivum* (Fig. 2B,C). Based on the phylogenetic relation between *B. distachyon* and *T. aestivum* and the other tested C_4_ grasses (Fig. 1), we would have anticipated differences in the cell wall composition between the two groups, C_3_ and C_4_ grasses, rather than within a group. Therefore, these differences in cellulose and hemicellulose content were the first indication that a phylogenetic relation might not directly refer to relatively high similarities in cell wall composition. Regarding lignin, highest amounts were found in biomass from *M.* x *giganteus* (Fig. 2A). The lignin content of *M.* x *giganteus* leaf biomass was significantly higher than in the two C_3_ grasses *B. distachyon* and *T. aestivum*. For stem biomass, differences in the lignin content resembled the phylogenetic relation only between the C_4_ grasses and *B. distachyon* as we determined significantly higher lignin content in *M.* x *giganteus* and *Z. mays* than in *B. distachyon*, whereas the lignin content of *T. aestivum* was not significantly different from the C_4_ grasses. A lignin content of 24% in *M.* x *giganteus* stem biomass, which was 40% higher than in *B. distachyon* stem biomass, marked the highest value in all tested samples (Fig. 2A). Apart from cellulose crystallinity that directly effects enzymatic saccharification [16]–[18], lignin is considered a major cell wall component, which reduces saccharification efficiency of biomass [19], [20]. Therefore, modification or reduction of the lignin content might improve saccharification efficiency, especially in *M.* x *giganteus* with its relatively high lignin content. The reduction of lignin biosynthesis by RNA interference, which in *S. officinarum* [47] and *Populus tomentosa* (Chinese white poplar) [48] correlated with reduced recalcitrance of the cell wall, might represent a promising approach. However, strategies that affect lignin biosynthesis should be accompanied by experiments that test putative alterations in plant defense to invading pathogens because lignin has a critical role in establishing disease resistance [49].

### Non-cellulosic monosaccharide composition

**Figure 3:**
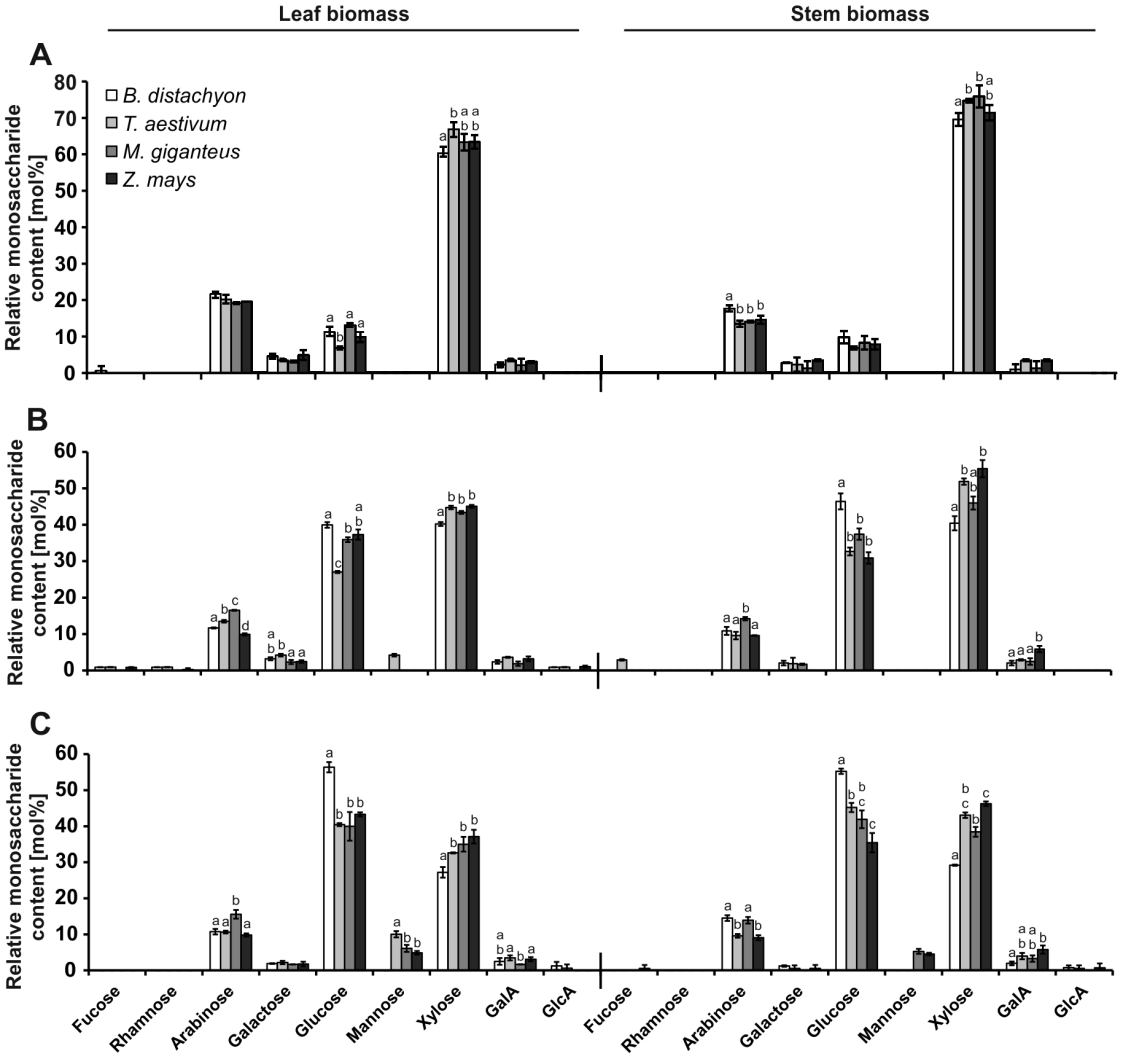
Relative non-cellulosic monosaccharide composition of leaf and stem biomass. Cell wall extracts from senesced, dry, and milled leaf and stem biomass samples were used. Non-cellulosic monosaccharide composition determined by HPAEC-PAD from (**A**) untreated biomass, (**B**) biomass autoclaved in diluted sulfuric acid (1.75% (v/v)), and (**C**) biomass autoclaved in diluted sulfuric acid with subsequent enzymatic hydrolysis (Accellerase 1500 enzyme mixture). Letters a, b, c, and d indicate groups with *P*<0.05 by Tukey's test. Error bars represent SE, and *n* = 3. GalA: galacturonic acid, GlcA: glucuronic acid.

High-performance anion exchange chromatography with pulsed amperometric detection (HPAEC-PAD) facilitated a detailed analysis of the non-cellulosic monosaccharide composition of the dried leaf and stem biomass. As expected from secondary grass cell walls [25], xylose, glucose, and arabinose were the predominant monosaccharides (Fig. 3). After destarching and before biomass pretreatment, the relative monosaccharide content of all four tested plant species was similar (Fig. 3A). Slight aberrations were only determined in the glucose and xylose content in *T. aestivum* leaf biomass and in the arabinose and xylose content in *B. distachyon* stem biomass. However, these slight aberrations did not influence the overall observation that the ratio of arabinose, glucose, and xylose resembled previous cell wall studies of these plant species [25], [31], [42], [50]. Differences in cell wall composition can generally derive from differences in plant cultivation. In this regard, it has been shown that cultivation conditions could influence the process of maturation in *M.* x *giganteus* and *Z. mays* [51]–[53]. Hence, the composition of the cell wall may not be directly comparable between different studies. This supports the approach in our study where we compare cell wall composition of plant biomass that reached its final development stage from different plant species grown under the same condition, which contributes to a higher comparability of datasets between different plant species.

We then analyzed the non-cellulosic monosaccharide composition after application of two different methods of biomass pretreatment, i) thermo-chemical treatment with diluted sulfuric acid (1.75% (v/v)) and ii) with an additional application of an commercially available enzyme mixture (Accellerase 1500) after a previous diluted sulfuric acid treatment. The combination of a thermo-chemical pretreatment using diluted acid with a subsequent enzyme application is regarded as one of the most promising strategies to overcome biomass recalcitrance and release of fermentable carbohydrates [54].

In the leaf biomass of all analyzed plant species, we observed a shift in the glucose to xylose mol%-ratio from 1/7 before treatment to nearly 1/1 ratio after sulfuric acid and combined sulfuric acid and enzymatic treatment (Fig. 3), which was even stronger for *B. distachyon* leaf biomass after the combined pretreatment. Here, the glucose to xylose mol%-ratio shifted to almost 2/1 (Fig. 3C). For stem biomass, we observed a similar increased recalcitrance of glucose-containing hemicelluloses compared to xylose-containing, which was again most prominent for *B. distachyon*, where the glucose to xylose mol %-ratio shifted from about 1/7 before treatment (Fig. 3A) to 1/1 after sulfuric acid pretreatment (Fig. 3B) and to almost 2/1 after combined acid and enzyme pretreatment (Fig. 3C). In addition to the differences in the non-cellulosic monosaccharide composition observed for *B. distachyon*, we found additional significant differences especially for the arabinose content of *M.* x *giganteus* leaf and stem biomass, which was highest after both, sulfuric acid and combined sulfuric acid and enzymatic treatment compared to the other plant species, and for *Z. mays* stem biomass where the galacturonic acid content was highest after the two types of biomass treatment (Fig. 3B,C). These results revealed that the applied pretreatment methods favored the release of xylose rather than glucose from hemicelluloses. Our results confirmed previous studies where xylan, the main hemicellulose and main source of xylose, was better hydrolyzed during thermo-chemical biomass pretreatment than glucomannan, which mainly consists of glucose residues [55]–[57]. Because the relative non-cellulosic glucose content of almost all *B. distachyon* biomass samples after pretreatment was significantly higher than in all other analyzed species, which correlated with a significantly lower xylose content (Fig. 3A,B), a relatively efficient hydrolysis of xylan can be expected in *B. distachyon*.

### Rapid qualitative analysis of biomass saccharification by confocal lambda scanning

**Figure 4:**
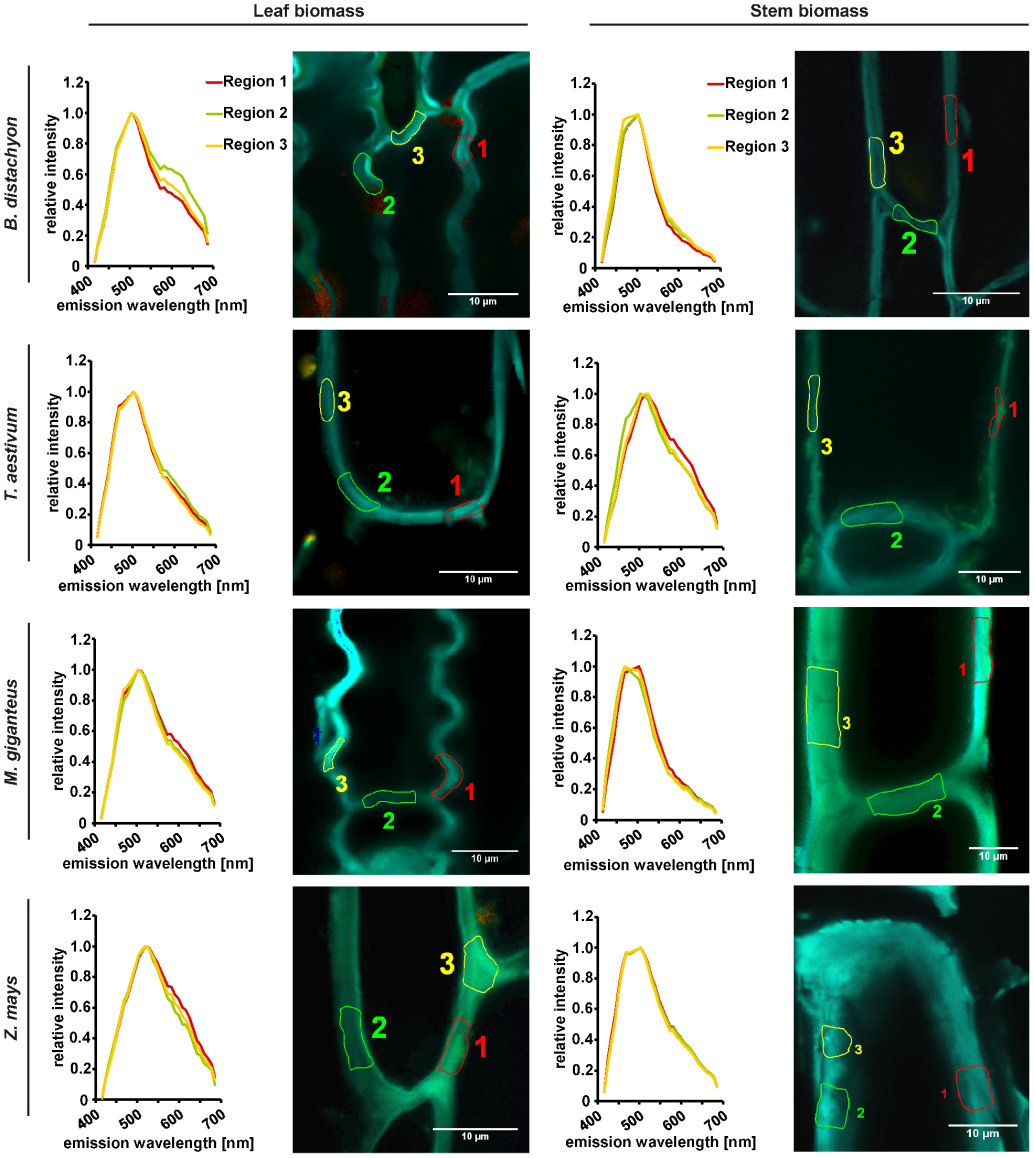
Rapid qualitative analysis of carbohydrate/lignin ratio in plant cell walls of untreated biomass by confocal lambda scanning. Cell wall particles from senesced, dry, and milled leaf and stem biomass samples were used for lambda scanning with a confocal laser-scanning microscope. Three defined cell wall regions in each leaf and stem sample were manually selected for measurement of emission spectra. Green fluorescence emitted from ferulic acid, cellulose, and additional carbohydrates, red fluorescence indicative for high lignin content. Micrographs are representative for each sample after evaluating at least five independent replicates.

**Figure 5:**
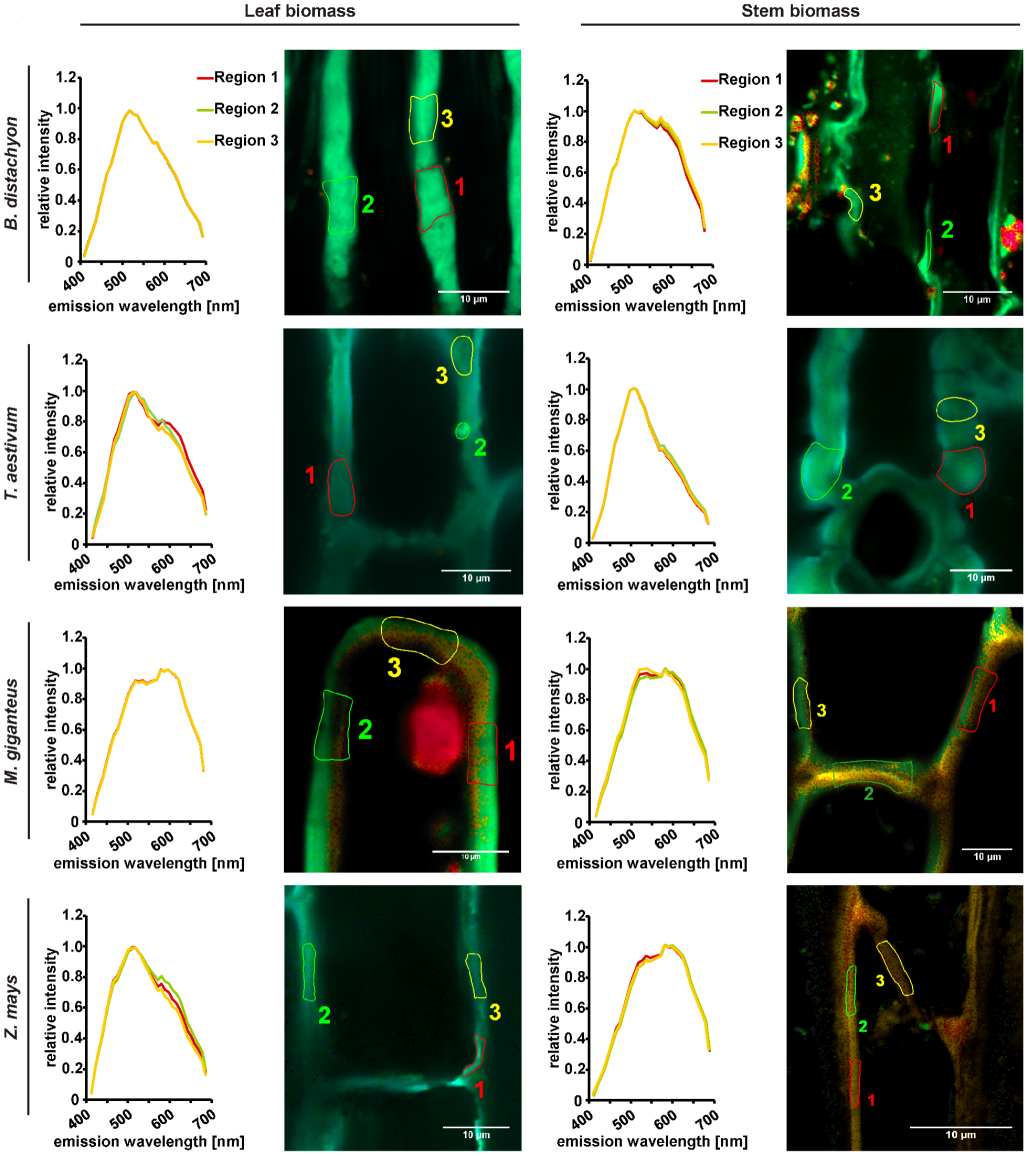
Rapid qualitative analysis of carbohydrate/lignin ratio in plant cell walls of thermo-chemical pretreated biomass by confocal lambda scanning. Cell wall particles from senesced, dry, and milled leaf and stem biomass samples were autoclaved in diluted sulfuric acid (1.75% (v/v)) before used for lambda scanning with a confocal laser-scanning microscope. Three defined cell wall regions in each leaf and stem sample were manually selected for measurement of emission spectra. Green fluorescence emitted from ferulic acid, cellulose, and additional carbohydrates, red fluorescence indicative for high lignin content. Micrographs are representative for each sample after evaluating at least five independent replicates.

**Figure 6:**
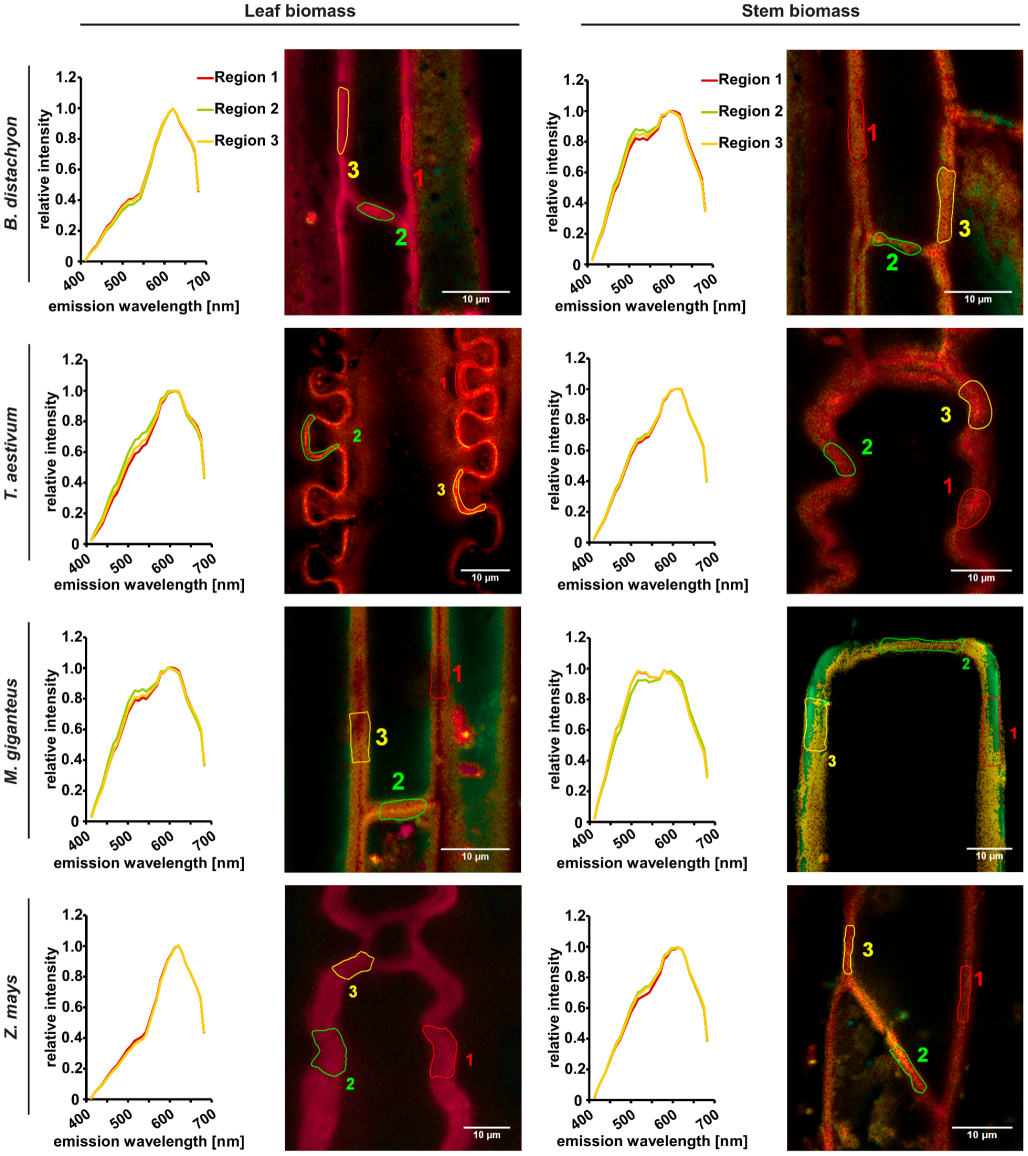
Rapid qualitative analysis of carbohydrate/lignin ratio in plant cell walls of thermo-chemical pretreated and hydrolyzed biomass by confocal lambda scanning. Cell wall particles from senesced, dry, and milled leaf and stem biomass samples were autoclaved in diluted sulfuric acid (1.75% (v/v)) with a subsequent enzymatic hydrolysis (Accellerase 1500 enzyme mixture) before used for lambda scanning with a confocal laser-scanning microscope. Three defined cell wall regions in each leaf and stem sample were manually selected for measurement of emission spectra. Green fluorescence emitted from ferulic acid, cellulose, and additional carbohydrates, red fluorescence indicative for high lignin content. Micrographs are representative for each sample after evaluating at least five independent replicates.

To test the application of a rapid qualitative analysis of biomass composition and saccharification after different methods of biomass pretreatment, we applied a lambda scan via confocal laser-scanning microscopy to the different tissue types of biomass deriving from all four analyzed plant species. The qualitative mapping of the plant cell walls is based on its auto-fluorescence during lambda scanning. The excitation of the plant biomass with the 405 nm diode laser induced the emission of green fluorescence, which mainly derives from bound ferulic acid in cell walls [58], [59] and carbohydrates; especially from cellulose [60], [61] as the most abundant polymer (Fig. 2). In this regard, a decrease in green fluorescence during pretreatment procedures as well as subsequent fermentation would not only indicate the hydrolysis of cellulose but also a reduction of cross-linked polysaccharides. The interdependency of these two processes is reflected by the fact that ester-linked ferulic acids at arabinose side chains of arabinoxylan are involved in the cross-linking of these cell wall polysaccharides and in forming ferulate-polysaccharide-lignin complexes that support cell wall cross-linking [62]. Therefore, the emitted green fluorescence can be seen as a marker for the recalcitrance of cell wall material to saccharification [63]. The parallel excitation of the plant cell wall material with 561 nm DPSS laser induced the emission of red-shifted fluorescence from *p*-hydroxyphenyl, guiacyl, and syringyl families of lignins with an accumulated wavelength of about 600 nm [35]. Manual selection of regions used for spectral analysis facilitated a precise identification of cell wall parts in the heterogeneous cell wall suspension, which minimized the interference of the measurement by background auto-fluorescence of the fermentation broth (Fig. 4). As expected from biomass without pretreatment, the leaf and stem cell walls of all plant species revealed a strong green auto-fluorescence peaking at about 500 nm, which indicated a relatively high content of cellulose and other carbohydrates as well as ferulic acid in cell walls (Fig. 4). The absolute strength of the green fluorescence overlaid the red fluorescence deriving from excited lignin, which was detected in parallel and indicated by a small shoulder formation at 600 nm mainly visible in leaf biomass samples (Fig. 4). After thermo-chemical pretreatment with diluted sulfuric acid, the predominant auto-fluorescence of the leaf cell walls from *B. distachyon*, *T. aestivum*, and *Z. mays* as well as *T. aestivum* stem cell walls was still green and peaked at about 500 nm. Hence, the efficiency of saccharification of this biomass was slightly lower compared to stem cell walls from *B. distachyon*, *Z. mays*, and especially *M.* x *giganteus*, where also leaf cell walls revealed a second peak at about 600 nm after spectral analysis (Fig. 5). This peak revealed a higher relative content of lignin due to hydrolysis of carbohydrates. Interestingly, the recalcitrance of leaf biomass was slightly higher than of stem biomass after thermo-chemical pretreatment. Based on previous reports [64], [65], we expected a higher recalcitrance of stem biomass, which would be mainly caused by a higher lignin content of stem biomass compared to leaf biomass (Fig. 2A). Therefore, other factors that determine biomass recalcitrance have to be considered. In this regard, the slightly higher hemicellulose content of leaf biomass samples than of stem biomass samples (Fig. 2C) might be involved as the content of hemicellulose affects cell wall digestibility due to a putatively higher cross-linking [62]. The combined biomass pretreatment with an initial diluted sulfuric acid and a subsequent enzymatic application resulted in a clearly improved saccharification of the cell walls. Auto-fluorescence from lignin components peaked at about 600 nm for almost all cell wall samples except the leaf and stem samples from *M.* x *giganteus* and the stem samples from *B. distachyon*, where a second, weaker but distinct 500 nm peak was present (Fig. 6), which indicated a relatively high carbohydrate content of the cell walls. Whereas increased recalcitrance of *M.* x *giganteus* biomass correlated with a relatively high lignin content in leaf and stem samples (Fig. 2A), we could not attribute a high lignin content to the relatively high recalcitrance of *B. distachyon* stem biomass because it was lowest in this measurement (Fig. 2A). Also the hemicellulose content, which can affect cell wall digestibility [62], was not significantly different from *T. aestivum* or *Z. mays* stem biomass that did not reveal an increased recalcitrance. This may indicate that cellulose crystallinity and/or ferulic acid-mediated cross-linking could be altered in *B. distachyon* stem cell walls. To analyze whether the observed recalcitrance of the leaf and stem cell walls from *M.* x *giganteus* and the stem cell walls from *B. distachyon* would be conserved during fermentation, we performed a lambda scan of sulfuric acid and enzymatic pretreated cell walls 72 h after start of fermentation. Whereas the degree of saccharification for the *B. distachyon* stem sample was comparable to the *B. distachyon* leaf sample and all *T. aestivum* and *Z. mays* samples, the *M.* x *giganteus* samples were more recalcitrant to carbohydrate hydrolysis compared to all other samples because spectral analysis still revealed a relatively strong 500 nm peak (Fig. S1). In general, the effectiveness of saccharification is not only dependent on the lignin content of the biomass but also on the level of hemicelluloses and cellulose. Comparing the cellulose und hemicelluloses level in our study with those reported by Xu et. al. [66], the dried *M.* x *giganteus* biomass that we used in this study revealed most similarities to those *Miscanthus* accessions that gave the lowest total sugar yield after diluted sulfuric acid biomass pretreatment with subsequent enzymatic hydrolysis. This might explain the relatively high degree of recalcitrance of *M.* x *giganteus* cell walls as indicated by the relatively strong emission of green fluorescence in confocal lambda scanning (Fig. 6).

### Determination of glucose content and ethanol production during fermentation

**Figure 7:**
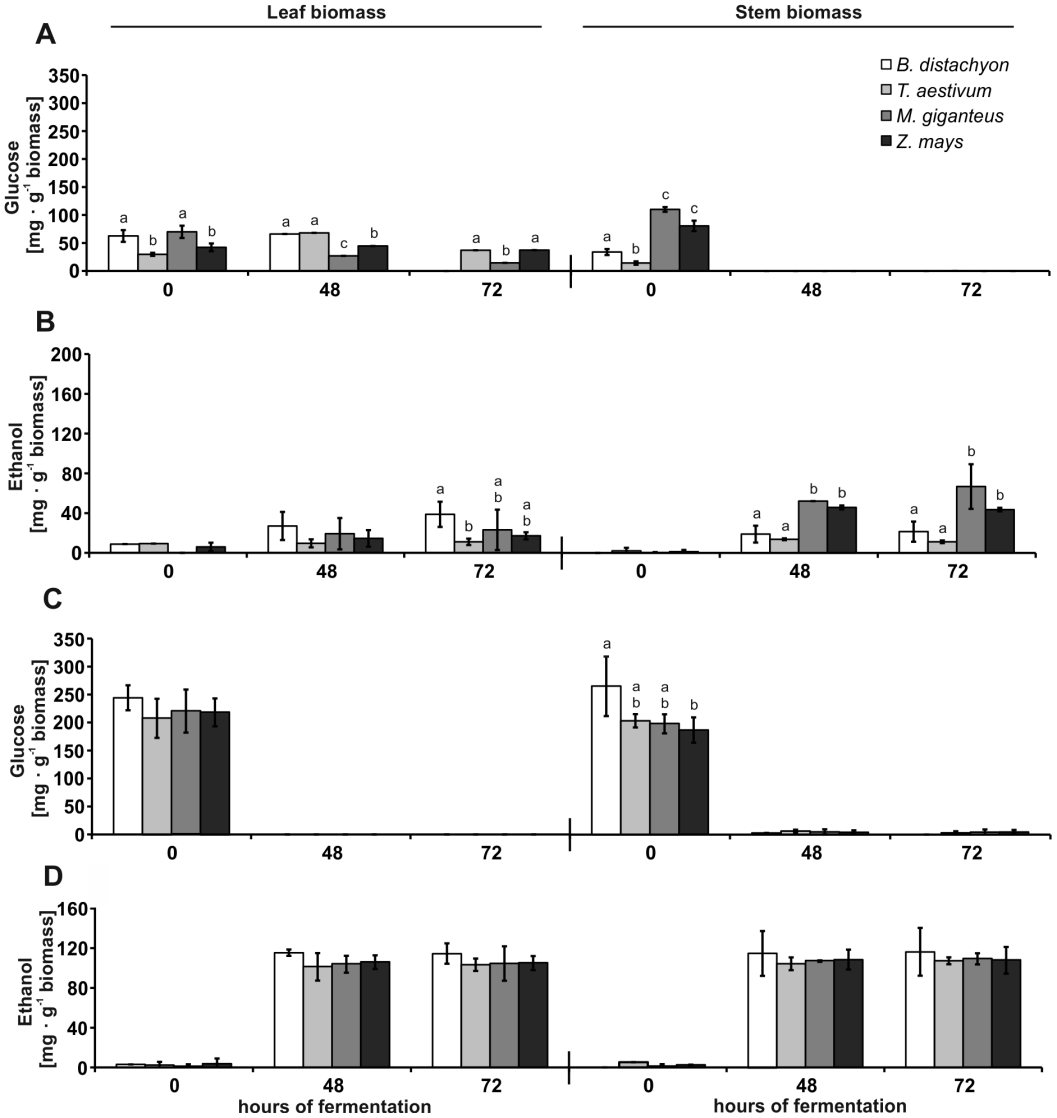
Glucose and ethanol concentration during fermentation of leaf and stem biomass. Glucose and ethanol concentrations in supernatants of the leaf and stem fermentation broth were determined by HPLC using a refractive index detector. Samples taken at the start of fermentation with *S. cerevisiae* and after 48 h and 72 h. (**A**) Glucose concentration and (**B**) ethanol concentration of biomass autoclaved in diluted sulfuric acid (1.75% (v/v)); (**C**) glucose concentration and (**D**) ethanol concentration of biomass autoclaved in diluted sulfuric acid with subsequent enzymatic hydrolysis (Accellerase 1500 enzyme mixture). Letters a, b, and c indicate groups with *P*<0.05 by Tukey's test. Error bars represent SE, and *n* = 3.

The quantification of the glucose content via HPLC in the pre-fermentation broth after biomass pretreatment resembled the observations that we previously made during lambda scanning. The degree of saccharification, which was indicated by a shift of the cell wall's auto-fluorescence from green to red (Figs. 4 and 6), correlated with the amount of released glucose; a low efficiency of saccharification and a low amount of released glucose after diluted sulfuric acid pretreatment (Figs. 5 and 7A), and an efficient saccharification with a high glucose concentration of the pre-fermentation broth after combined sulfuric acid and enzymatic biomass pretreatment (Figs. 6 and 7C). We also observed a correlation between the results of the spectral cell wall analysis and the HPLC analysis of glucose deriving from biomass that was only pretreated with diluted sulfuric acid. Those samples that showed the strongest shift of the auto-fluorescence spectrum from green to red also revealed the highest amount of released glucose. This was most prominent for stem biomass where *M.* x *giganteus* and *Z. mays* showed the most distinct green to red shift in the spectral cell wall analysis (Fig. 5), which resembled the three-fold and two-fold higher amount of hydrolyzed glucose compared to *B. distachyon* and *T. aestivum* (Fig. 5A; Tab. S1). Consequently, we detected a three-fold higher ethanol production for *M.* x *giganteus* and a two-fold higher ethanol production for *Z. mays* stem biomass compared to *B. distachyon* and *T. aestivum* (Fig. 7B; Tab. S1). Unlike stem biomass after diluted sulfuric acid treatment, we detected glucose in supernatants of the fermentation broth from leaf biomass even 48 h and 72 h after the start of fermentation (Fig. 7A). This indicated that the fermentation could be inhibited by compounds or cell wall components that might be only found in the leaf but not the stem where we did not detected an increased inhibition of fermentation. In general, furans formed by carbohydrate hydrolysis and phenolic monomers, which derive from lignin degradation, are known inhibitors of biomass fermentation with *S. cerevisiae* [67]. Apart from biochemical approaches to reduce fermentation inhibitors [68] or the generation of *S. cerevisiae* strains with improved resistance to inhibitors [69], different process technologies like continuous fermentation have been discussed to reduce the problem of inhibition [70]. The increase in the released glucose amount of sulfuric acid pretreated leaf biomass from *T. aestivum* between 0 h and 48 h after the start of fermentation (Fig. 7A) could indicate an ongoing saccharification process. Under these pretreatment conditions, the stem biomass of the C_4_ grasses *M.* x *giganteus* and *Z. mays* revealed the best ethanol production performance (Fig. 5), which was mainly based in relatively high glucose yield of 11% and 8% of the total biomass, respectively (Tab. S1). The differences in saccharification efficiency and ethanol production, which we observed with biomass that was only pretreated with diluted sulfuric acid, were almost equalized when an additional enzymatic pretreatment followed the initial acid pretreatment. On average, results from leaf biomass revealed a four- to seven fold higher biomass to glucose conversion efficiency due to enzymatic hydrolysis for the C_3_ grasses and a three- to five-fold higher conversion efficiency for the C_4_ grasses (Fig. 7C; Tab. S1). The increased amount of produced ethanol (three-fold for *B. distachyon*, ten-fold for *T. aestivum*, and almost six-fold for *M.* x *giganteus* and *Z. mays*) resembled the improved saccharification efficiency (Tab. S1). Interestingly, we did not detect glucose in the supernatant of the fermentation broth from leaf biomass with combined acid and enzymatic pretreatment (Fig. 7C) as we did after single acid treatment (Fig. 7A). This not only indicated a more efficient hexose release due to the pretreatment before fermentation but probably also less putative fermentation inhibitors. Hence, a comparative compound analysis of the differentially pretreated leaf biomass might support the identification of fermentation inhibitors, especially from *T. aestivum* leaf biomass, where our data suggest a higher level of inhibitors than in the other three plant species. Our observation that the enzyme mixture may reduce the concentration of putative fermentation inhibitors would support their identification. Putative fermentation inhibitors might be tested for the susceptibility to degradation by the enzyme mixture used for saccharification. Comparing leaf and stem biomass after combined acid and enzymatic pretreatment, we did not determine major differences in saccharification or ethanol production (Fig. 7C,D). Within stem biomass samples, only *B. distachyon* stem biomass showed an increased amount of released glucose, which was significantly higher than in *Z. mays*, but did not result in a significantly higher ethanol production (Fig. 7D). The amounts of produced ethanol from *M.* x *giganteus* of 105–110 mg·g^−1^ dry biomass that we determined for diluted acid and enzymatically pretreated biomass (Tab. S1) were in the range of ethanol amounts (100–110 mg·g^−1^ dry biomass) that were yielded in previous *M.* x *giganteus* studies where different methods of biomass pretreatment were applied, like the usage of ammonia fiber expansion or the ethanol organosolv process [71]–[73]. For *Z. mays* biomass, our ethanol yields after diluted acid and enzymatic pretreatment of 106–109 mg ethanol·g^−1^ dry biomass outcompeted the ethanol yields of 20–100 mg·g^−1^ dry biomass as reported in previous studies with alternative pretreatment methods [74]–[77]. Only for *T. aestivum*, we yielded less ethanol than in a comparable study with a dilute acid and enzymatic pretreatment [46]. The higher ethanol yield in that study could be explained by the usage of a recombinant *Escherichia coli* strain and overliming of the biomass hydrolyzates, which seems to be more effective in *T. aestivum* biomass fermentation than the usage of *S. cerevisiae* without overliming.

### Principal component analysis

**Figure 8:**
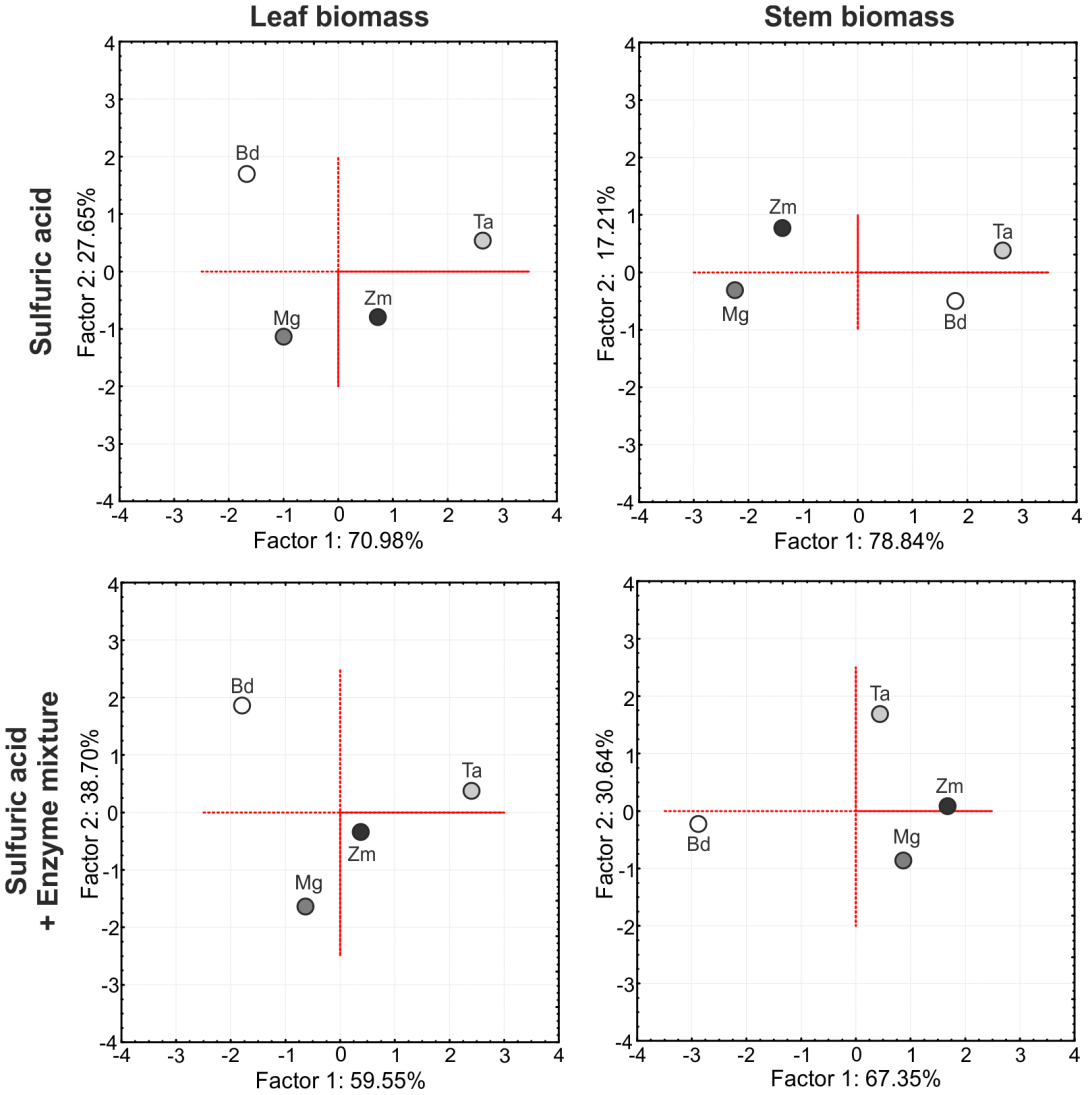
Principal components analysis of leaf and stem biomass after different pretreatment methods. Two-factor principal component analysis on the basis of six parameters defining efficiency of lignocellulosic ethanol production. Bd: *B. distachyon*, Mg: *M.* x *giganteus*, Ta: *T. aestivum*, Zm: *Z. mays*.

To evaluate and summarize the potential of *B. distachyon* as model plant for the production of ethanol by fermentation of senesced and dried leaf and stem biomass, we performed separated two-factored principal component analyses (PCA) of stem and leaf biomass with a thermo-chemical diluted sulfuric acid pretreatment and the combined pretreatment with diluted sulfuric acid followed by enzymatic hydrolysis. The calculation of the PCA and factor analysis was based on six different parameters that comprised cell wall composition, saccharification efficiency, and ethanol production from pretreated biomass of *B. distachyon*, *T. aestivum*, *M.* x *giganteus*, and *Z. mays*. A parallel analysis of multiple parameters would allow a more precise prediction of possible similarities than an evaluation of a single parameter or simple combinations as shown in *Z. mays* [78]. The PCA revealed that *B. distachyon* is most suitable as model for the process of lignocellulosic ethanol production from stem biomass of the tested *T. aestivum* line after thermo-chemical biomass pretreatment. Previously, *B. distachyon* was also described as a suitable model to study modifications of arabinoxylan structure and biosynthesis that could be applied on monocot energy crops for saccharification improvements [79]. However, based on our PCA results, *B. distachyon* would neither qualify as a good model for the process of lignocellulosic ethanol production from stem biomass of the tested crop lines after combined thermo-chemical and enzymatic biomass pretreatment nor as a good model for lignocellulosic ethanol production from leaf biomass independent of the applied method for biomass pretreatment (Fig. 8). A constant, direct correlation between phylogenetic relation and similarity in lignocellulosic ethanol production was only found for the C_4_ grasses *M.* x *giganteus* and *Z. mays*, which was independent of the applied biomass pretreatment method and type of biomass (Fig. 8). In contrast to its phylogenetic relation, *T. aestivum* revealed a higher similarity in lignocellulosic ethanol production to the C_4_ grasses *M.* x *giganteus* and *Z. mays* than to the C_3_ model grass *B. distachyon* especially in the two scenarios with a combined thermo-chemical and enzymatic biomass pretreatment (Fig. 8). This may reflect previous observations where even within a plant species, *Z. mays*, strong differences in cell wall composition and saccharification were reported from different cultivars [45]. Therefore, it has to be generally considered that phylogenetic relation of plant species does not directly indicate a good suitability as model for lignocellulosic ethanol production. The PCA analysis also showed that the degree of similarity in lignocellulosic ethanol production can be altered by the application of different biomass pretreatment methods. This was most obvious for stem biomass of *T. aestivum* where the combined acidic and enzymatic pretreatment increased similarity in ethanol production to *Z. mays* and *M.* x *giganteus* but broke the relatively high similarity to *B. distachyon* compared to a single acidic biomass pretreatment (Fig. 8). For stem biomass, we also determined a difference of the principal component (PC) loadings associated with the PCA after different types of biomass pretreatment. Whereas the variable “cellulose amount” contributed most to the separation of factor 1 and “lignin amount” to factor 2 after single acidic biomass pretreatment, the PC loading pattern changed to “ethanol production” for factor 1 and “cellulose amount” for factor 2 after combined acidic and enzymatic pretreatment (Tab. S2). For leaf biomass, “cellulose amount” was the variable with the highest contribution to the separation of factor 1 and “ethanol production” of factor 2, which was independent of the applied biomass pretreatment method (Tab. S2). Except for stem biomass after single acidic pretreatment, the variable “glucose amount from hemicellulose fraction” revealed a constantly low contribution to the separation of the two factors associated with the PCA.

Our PCA results suggest that predictions for the suitability of model plant for lignocellulosic ethanol production can strongly differ among species, tissue type and applied pretreatment method. Similar observations were previously made for possible predictions of cell wall digestibility in *P. virgatum* and *Andropogon gerardii* (big bluestem) [65].

In general, PCA is a method that can provide a fast an easy overview of the suitability of a plant species to be considered as model for another plant species. This kind of analysis can easily be extended with additional plant species and parameters, or plant species parameters can be modified, replaced or reduced if the focus of comparative analysis is on different traits than saccharification and ethanol production from leaf and stem biomass as in our study of the four plant species.

## Conclusions

The suitability of *B. distachyon* as model plant for lignocellulosic ethanol production from leaf and stem biomass was analyzed in comparative study with the two major crops from temperate climate, *T. aestivum* and *Z. mays*, and the emerging energy crop *M.* x *giganteus*. The model qualities of *B. distachyon* were best for stem biomass from *T. aestivum* after thermo-chemical biomass pretreatment whereas model qualities for leaf biomass were only limited for all tested crop lines. The ethanol yield from stem biomass ranging from 108 mg·g^−1^ dry biomass for *T. aestivum* to 117 mg·g^−1^ dry biomass for *B. distachyon* after combined thermo-chemical and enzymatic pretreatment was generally higher than from leaf biomass for all tested plant species. The results revealed that phylogenetic relation does not automatically reflect suitability as model for biomass conversion as the C_3_ grass *B. distachyon* offered promising qualities as model plant for biomass from the phylogenetically related crop *T. aestivum* only in one out of four approaches.

## Supporting Information

Figure S1 **Rapid qualitative analysis of carbohydrate/lignin ratio in plant cell walls of thermo-chemical pretreated and hydrolyzed biomass after fermentation by confocal lambda scanning.** Cell wall particles from senesced, dry, and milled leaf and stem biomass samples were autoclaved in diluted sulfuric acid (1.75% (v/v)) with a subsequent enzymatic hydrolysis (Accellerase 1500 enzyme mixture). After 72 h of fermentation, remaining cell wall particles were used for lambda scanning with a confocal laser-scanning microscope. Three defined cell wall regions in each leaf and stem sample were manually selected for measurement of emission spectra. Green fluorescence emitted from ferulic acid, cellulose, and additional carbohydrates, red fluorescence indicative for high lignin content. Micrographs are representative for each sample after evaluating at least five independent replicates.
(TIFF)

Table S1 **Overview of glucose saccharification and ethanol production of leaf and stem biomass with different pretreatment methods.** Biomass pretreatment: acid, biomass autoclaved in diluted sulfuric acid (1.75% (v/v)); acid+enzymes, biomass autoclaved in diluted sulfuric acid (1.75% (v/v) with subsequent enzymatic hydrolysis (Accellerase 1500 enzyme mixture). Values in brackets represent SE, and *n* = 3.
(TIFF)

Table S2 **Principal component (PC) loadings associated with the PC analysis.** PC loadings indicate importance of each variable in accounting for the variability of factor 1 and 2. Biomass pretreatment: sulfuric acid, biomass autoclaved in diluted sulfuric acid (1.75% (v/v)); sulfuric acid+enzyme mixture, biomass autoclaved in diluted sulfuric acid (1.75% (v/v) with subsequent enzymatic hydrolysis (Accellerase 1500 enzyme mixture).
(TIFF)
