# Supplementary figures and images for: Phylogeny in Defining Model Plants for Lignocellulosic Ethanol Production: A Comparative Study of Brachypodium distachyon, Wheat, Maize, and Miscanthus x giganteus Leaf and Stem Biomass
Source: PLoS One. 2014 Aug 18;9(8):e103580. doi: 10.1371/journal.pone.0103580 (PMC4136770; doi:10.1371/journal.pone.0103580)

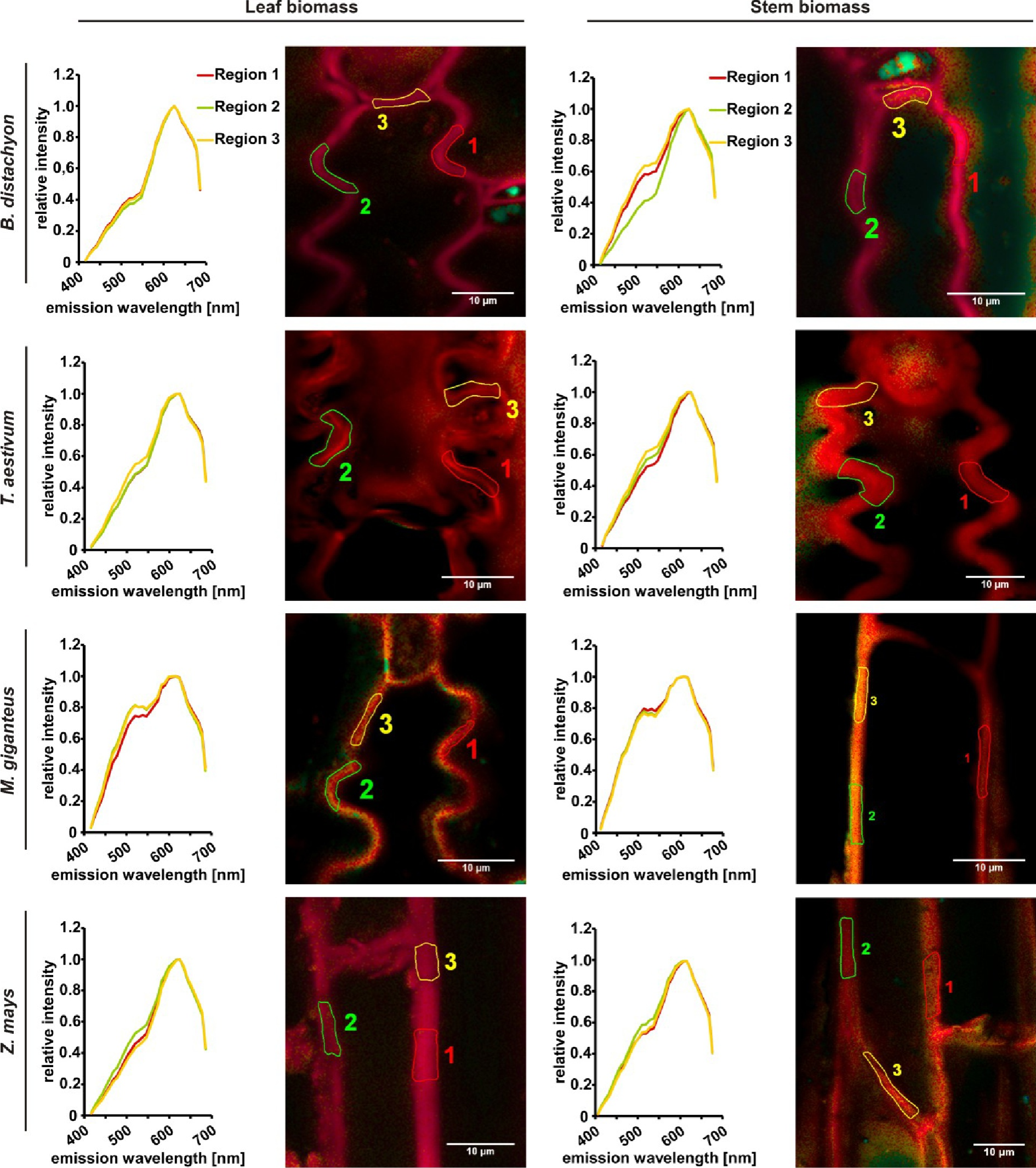

Supplement: Figure S1 — Rapid qualitative analysis of carbohydrate/lignin ratio in plant cell walls of thermo-chemical pretreated and hydrolyzed biomass after fermentation by confocal lambda scanning. Cell wall particles from senesced, dry, and milled leaf and stem biomass samples were autoclaved in diluted sulfuric acid (1.75% (v/v)) with a subsequent enzymatic hydrolysis (Accellerase 1500 enzyme mixture). After 72 h of fermentation, remaining cell wall particles were used for lambda scanning with a confocal laser-scanning microscope. Three defined cell wall regions in each leaf and stem sample were manually selected for measurement of emission spectra. Green fluorescence emitted from ferulic acid, cellulose, and additional carbohydrates, red fluorescence indicative for high lignin content. Micrographs are representative for each sample after evaluating at least five independent replicates. (TIFF) [file pone.0103580.s001.tiff]

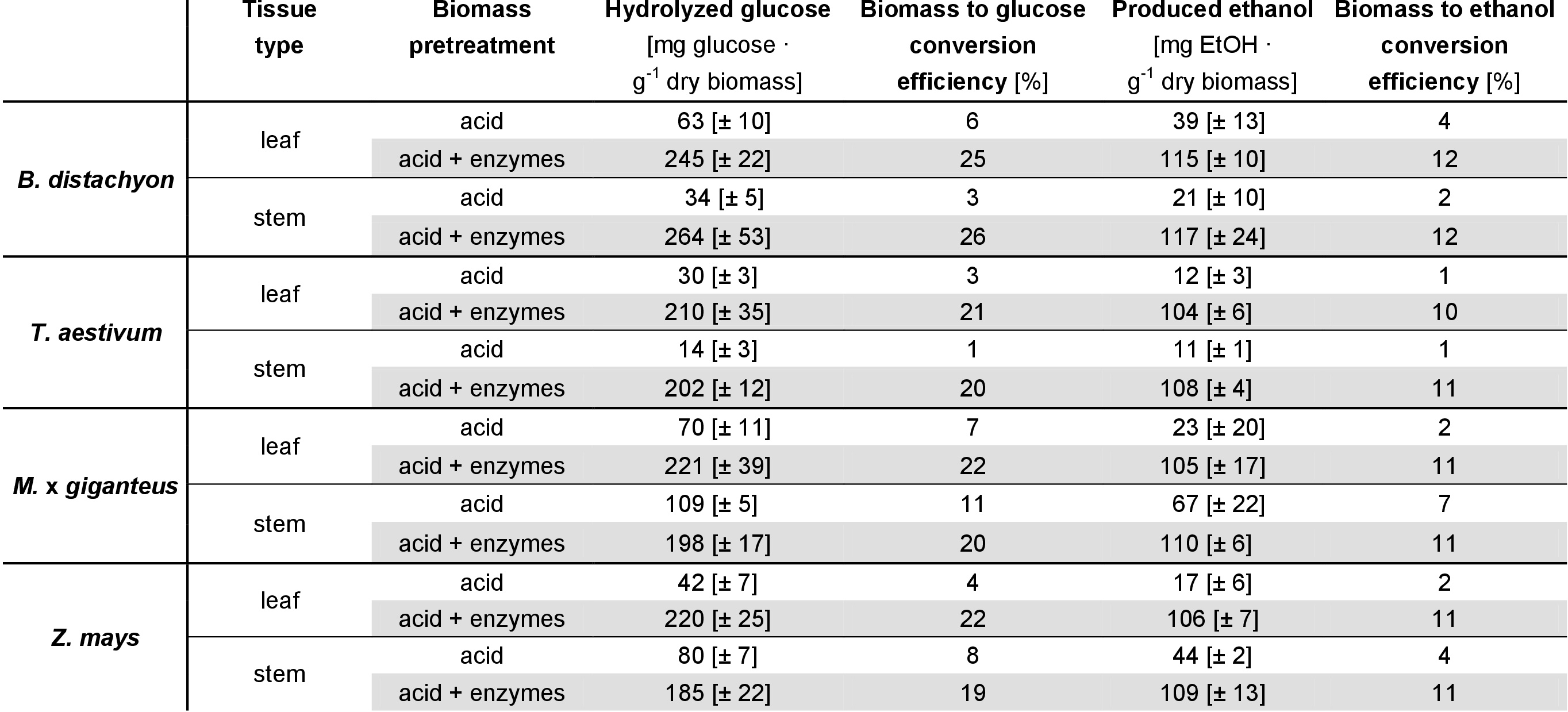

Supplement: Table S1 — Overview of glucose saccharification and ethanol production of leaf and stem biomass with different pretreatment methods. Biomass pretreatment: acid, biomass autoclaved in diluted sulfuric acid (1.75% (v/v)); acid+enzymes, biomass autoclaved in diluted sulfuric acid (1.75% (v/v) with subsequent enzymatic hydrolysis (Accellerase 1500 enzyme mixture). Values in brackets represent SE, and n = 3. (TIFF) [file pone.0103580.s002.tiff]

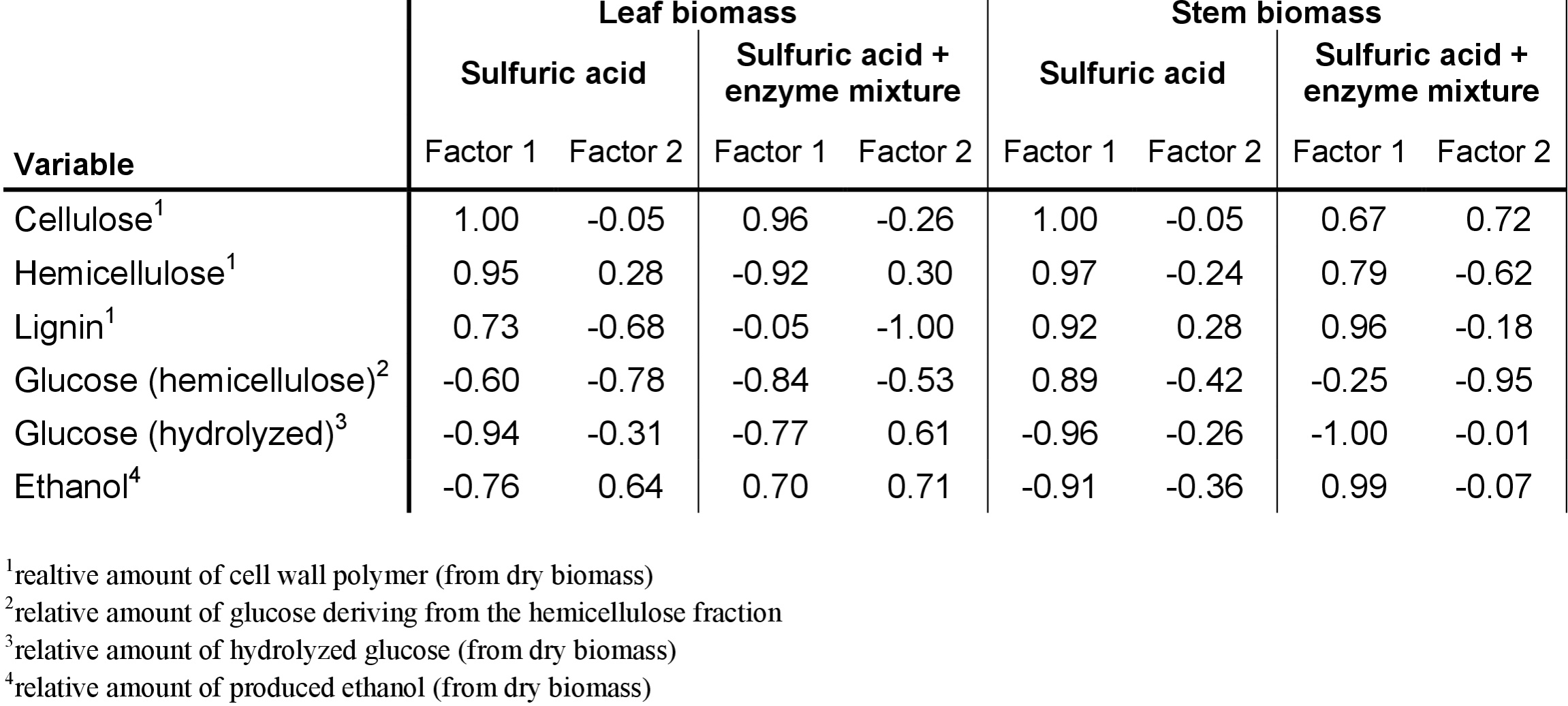

Supplement: Table S2 — Principal component (PC) loadings associated with the PC analysis. PC loadings indicate importance of each variable in accounting for the variability of factor 1 and 2. Biomass pretreatment: sulfuric acid, biomass autoclaved in diluted sulfuric acid (1.75% (v/v)); sulfuric acid+enzyme mixture, biomass autoclaved in diluted sulfuric acid (1.75% (v/v) with subsequent enzymatic hydrolysis (Accellerase 1500 enzyme mixture). (TIFF) [file pone.0103580.s003.tiff]
